# Regulation of Bone Morphogenetic Protein Signaling by ADP-ribosylation*

**DOI:** 10.1074/jbc.M116.729699

**Published:** 2016-04-21

**Authors:** Yukihide Watanabe, Panagiotis Papoutsoglou, Varun Maturi, Yutaro Tsubakihara, Michael O. Hottiger, Carl-Henrik Heldin, Aristidis Moustakas

**Affiliations:** From ‡ Ludwig Cancer Research, Science for Life Laboratory, Uppsala University, SE-751 24 Uppsala, Sweden,; the § Department of Molecular Mechanisms of Disease, University of Zurich, 8057 Zurich, Switzerland, and; the ¶ Department of Medical Biochemistry and Microbiology, Science for Life Laboratory, Uppsala University, SE-751 23 Uppsala, Sweden

**Keywords:** ADP-ribosylation, bone morphogenetic protein (BMP), signal transduction, SMAD transcription factor, transcription

## Abstract

We previously established a mechanism of negative regulation of transforming growth factor β signaling mediated by the nuclear ADP-ribosylating enzyme poly-(ADP-ribose) polymerase 1 (PARP1) and the deribosylating enzyme poly-(ADP-ribose) glycohydrolase (PARG), which dynamically regulate ADP-ribosylation of Smad3 and Smad4, two central signaling proteins of the pathway. Here we demonstrate that the bone morphogenetic protein (BMP) pathway can also be regulated by the opposing actions of PARP1 and PARG. PARG positively contributes to BMP signaling and forms physical complexes with Smad5 and Smad4. The positive role PARG plays during BMP signaling can be neutralized by PARP1, as demonstrated by experiments where PARG and PARP1 are simultaneously silenced. In contrast to PARG, ectopic expression of PARP1 suppresses BMP signaling, whereas silencing of endogenous PARP1 enhances signaling and BMP-induced differentiation. The two major Smad proteins of the BMP pathway, Smad1 and Smad5, interact with PARP1 and can be ADP-ribosylated *in vitro*, whereas PARG causes deribosylation. The overall outcome of this mode of regulation of BMP signal transduction provides a fine-tuning mechanism based on the two major enzymes that control cellular ADP-ribosylation.

## Introduction

The mechanisms of regulation of signaling pathways in the TGF-β family present significant complexity and are of paramount biological importance because they explain how these pathways mediate physiological developmental processes and how their activities malfunction under pathological conditions (1–3). Among the 33 genes that are enlisted in the TGF-β family, the largest group is the bone morphogenetic protein (BMP)^4^ subfamily. BMPs have established functions in the control of tissue growth and differentiation acting during embryonic development and in adulthood, as mediators of homeostatic regulation (4–6). A hallmark function of many of the BMP members is the differentiation of mesenchymal progenitors into osteoblasts that will produce mature osteocytes and into chondroblasts that will generate chondrocytes during the development and remodeling of cartilage and bone (7). For example, pluripotent mouse C2C12 myoblasts can differentiate into osteoblasts in response to many BMP ligands (8). During osteoblast differentiation, genes such as *ID1*, the negative regulator of basic helix-loop-helix transcription factors, and *ALP* (*alkaline phosphatase*) are transcriptionally induced by BMP signaling (9, 10).

BMP signaling proceeds when ligands associate with their type I and type II serine/threonine kinase receptors, whereby the type II receptor kinase phosphorylates specific serine residues in the type I receptor and changes its conformation, leading to activation of the protein kinase in the type I receptor (11, 12). The activated type I receptor then phosphorylates Smad1, Smad5, and Smad8, the three receptor-activated Smads (R-Smads), in their C-terminal Ser-Xaa-Ser motifs, which then associate with Smad4, the common mediator Smad (11, 12). In this manner, activated complexes of R-Smad and Smad4 bind to chromatin and regulate expression of target genes such as *ID1* and *ALP*. The ligand-bound oligomeric receptor complex also activates members of the MAPK family and their upstream protein kinases, leading to a more integrated regulation of the gene targets of the BMP pathway (12, 13).

The BMP pathway is controlled by a series of negative regulatory mechanisms that ensure proper duration, magnitude of response, and differential kinetics depending on cell type and biological context (1–3). Major negative regulators of BMP signaling are the inhibitory Smads (I-Smads), Smad6 and Smad7, whose genes directly respond to BMP-dependent Smad and MAPK signaling, thus forming negative feedback loops (11, 12). The I-Smads can interact with the BMP type I receptors and competitively inhibit R-Smad phosphorylation, bind to Smad4, block the transcriptional activity of Smad complexes, and also recruit ubiquitin ligases to the type I receptors to promote their internalization and degradation (1, 2).

An alternative mechanism of negative control of TGF-β signaling pathways involves the post-translational modification of Smad proteins by members of the intracellular ADP-ribosyltransferases structurally related to diphtheria toxin (ARTDs). The best characterized member of the ARTD family is ARTD1, also called poly-(ADP-ribose) polymerase-1 (PARP1) (14–16). PARP1 can be activated during damage of DNA from chemical agents or radiation and initiates the DNA damage response that mends the lesions produced on DNA. In addition, PARP1 can ADP-ribosylate transcription and chromatin remodeling factors as well as histones, thus controlling the processes of transcription and DNA methylation (14, 15). PARP1 exhibits complex regulation of its catalytic activity, and it can also ribosylate itself in an autocatalytic manner (17). In addition, substrates that are ADP-ribosylated by PARP1 also lose their ADP-ribose modification by the sequential action of the enzyme poly-(ADP-ribose) glycohydrolase (PARG) that cleaves poly-(ADP-ribose) chains (18) and ADP-ribosylhydrolase 3 or macrodomain-containing proteins (*e.g.* MacroD1) that hydrolyze mono-ADP-ribose from modified substrates (19). Accordingly, transcription is regulated by the balanced action of PARP1 and PARG, which dynamically control the degree of ADP-ribosylation of chromatin-bound proteins (20). PARG is encoded by a single gene, which gives rise to different isoforms. The longer isoform is a nuclear 111-kDa protein, whereas the shorter 102-, 99-, and 60-kDa isoforms are predominantly cytoplasmic (21).

Smad3 and Smad4 binding to chromatin is inhibited after ADP-ribosylation of their conserved N-terminal Mad homology 1 (MH1) domain (22). ADP-ribosylation of Smad3 and Smad4 is catalyzed by the nuclear enzymes PARP1 and its sibling PARP2, which associate with each other and with the Smad proteins in the nucleus (22, 23). ADP-ribose chains are removed from Smad3 and Smad4 by the action of PARG, which plays a positive regulatory role during TGF-β signaling (23). PARP1 acting in T lymphocytes participates in the transcriptional repression of the receptor genes for TGF-β (24). This finding is in agreement with the binding of PARP1 in the promoter sequences of the TGF-β type II receptor gene, as analyzed in breast cancer cells (25). In agreement with the negative regulation of TGF-β signaling by PARP1, prostate tumors developing in a mouse carrying complete loss of function mutation of PARP1 revealed enhanced epithelial-mesenchymal transition caused by enhanced TGF-β signaling in the prostate carcinoma cells (26), which corroborates our original findings whereby PARP1 impacted the mesenchymal transition of mammary epithelial cells (22). On the other hand, the functions of TGF-β in vascular smooth muscle cells can be positively affected by the activity of PARP1 (27). Despite this knowledge, the impact of members of the ARTD family on BMP signaling and BMP-specific Smad proteins remains unknown.

In this article, we address the question of regulation of BMP signaling by ADP-ribosylation. We report that PARG positively regulates BMP signaling and osteoblast differentiation, whereas PARP1 is a negative regulator. A corollary of this functional importance of the two enzymes that control ADP-ribosylation is the formation of protein complexes between R-Smads of the BMP pathway and PARG and PARP1, as revealed by immunoprecipitation and proximity ligation assays (PLA). In addition, Smad1 and Smad5 can be ADP-ribosylated by PARP1, and PARG removes the ADP-ribose chains from these Smads. The new evidence establishes ADP-ribosylation as a widespread regulatory mechanism of Smad proteins in the TGF-β and BMP families.

## Experimental Procedures

### 

#### 

##### Cell Culture and Transfections

HEK293T cells were cultured according to protocols from the American Type Culture Collection (LGC Standards AB, Borås, Sweden). Human immortalized keratinocytes HaCaT were cultured as described previously (28). C2C12 mouse myoblasts and C2C12 cells stably transfected with BMP-responsive element (BRE) construct (named as C2C12-BRE-luc, a kind gift of P. ten Dijke, Leiden University Medical Center, Leiden, The Netherlands) were cultured in DMEM supplemented with 10% FBS and penicillin-streptomycin. PARP1 knock-out mouse embryonic fibroblasts (PARP1^−^ MEFs) were kindly provided by J. Ménissier-de Murcia (University of Strasbourg, Strasbourg, France) (29). Transient transfections of cells were performed using FuGENE HD (Roche) or Lipofectamine 2000 (Life Technologies) according to the manufacturer's protocols. siRNA oligonucleotides were purchased from Dharmacon/Thermo Fischer Scientific, as pools or individual pure molecules. Transfection of siRNA oligonucleotides (20–25 nm) targeting human PARP1 (Dharmacon ONTARGETplus SMARTpool L-006656-00, individuals LU-006656-03, J-006656-06, siPARP1-1, and J-006656-08, siPARP1-3), mouse PARP1 (Dharmacon ONTARGETplus SMARTpool L-040023), human PARG (Dharmacon ON-TARGETplus SMARTpool L-011488-00 individuals, LU-011488-00, J-011488-05, siPARG-1, J-011488-07, and siPARG-3) or non-targeting control pool (Dharmacon ONTARGETplus Non-targeting pool D-001810-10), was performed using siLentfect (Bio-Rad) transfection reagent. The cells were transfected a single time or two times with a retransfection after 24 h for totally 36 or 48 h and cultured in DMEM containing 0.1, 1, or 10% FBS prior to stimulations and cell-based assays.

##### Growth Factors, Plasmids, and Other Reagents

Recombinant mature human BMP7 was a gift from K. Sampath (Genzyme-Sanofi). The dose used for BMP7 was 5 ng/ml, unless indicated otherwise. Human mature BMP2 was a gift of H. F. Lodish (Whitehead Institute for Biomedical Research, MIT, Cambridge, MA). Recombinant mature human BMP4 and TGF-β1 were bought from PeproTech EC Ltd. (London, UK). All growth factors were dissolved in vehicle consisting of 4 mm HCl, 0.1% (w/v) fatty acid-free bovine serum albumin. The second generation analog of dorsomorphin and highly selective small molecule inhibitor of BMP type I receptors, dorsomorphin homolog 1, 4-[6-[4-(1-methylethoxy)phenyl]pyrazolo[1,5-a]pyrimidin-3-yl]-quinoline, was synthesized by Ludwig Cancer Research. The PARP1 inhibitors 3-aminobenzamide (3-AB) and PJ34 were purchased from Sigma-Aldrich, and Axxora, LLC/ENZO Life Sciences, respectively. H_2_O_2_ and Coomassie Brilliant Blue R250 were obtained from Merck, whereas β-NAD^+^ was bought from Sigma-Aldrich. High purity recombinant PARP1 and PARG (20,000 units/mg, 0.1 μg/ml) isolated from insect cells after baculoviral infection were bought from Axxora, LLC/ENZO Life Sciences.

The WT BRE-luc reporter (BRE)_2_-luc and pCMV-β-galactosidase used for normalization of transfection efficiency have been described before (30). The mutant BRE-luc reporter (BRE)_2_-luc#211 that shows very weak binding of Smad proteins and is weakly activated by BMP signaling was kindly provided by P. ten Dijke (Leiden University Medical Center, Leiden, The Netherlands) (10). Expression vectors pcDNA3-FLAG-Smad1, pcDNA3-FLAG-Smad3, pcDNA3-FLAG-Smad4, pcDNA3-FLAG-Smad5, and pcDNA3-myc-PARP1 have also been previously described (22, 23, 30). Expression vector pCS2-Myc-PARG and the empty vector pCS2 were kindly provided by P. Caiafa (Sapienza University, Rome, Italy). Glutathione *S*-transferase fusions of Smads, GST-Smad1, and GST-Smad5 were constructed by transfer from the pcDEF3 vector to pGEX-4T-1. Smad1 MH1 (residues 1–132) was amplified using *Pfu* polymerase and was inserted into pGEX-4T-1 using the primer pair 5′-CGCGGATCCAATGTGACAAGTTTATTTTCC-3′ and 5′-CCGCTCGAGTCAGCTTTCTACTCTCTTATAGTG-3′.GST-Smad1 MH1 mutants (K53A, K53R, and EELE to QQLQ) were made by introducing a mutation in GST-Smad1 MH1 to change Lys^53^ to Ala with double-stranded primer pair (only the sense strand is shown, and the mutation is indicated by lowercase and bold letters) 5′-GCCATGGAGGAACTGGAA**gc**GGCCTTGAGCTGCCCAGGG-3′, Lys^53^ to Arg using primer pair 5′-GCCATGGAGGAACTGGAAA**g**GGCCTTGAGCTGCCCAGGG-3′, and ^49^EELE to QQLQ using primer pair 5′-GAAAAAGAAAGGTGCCATG**c**AG**c**AACG**c**AAAAGGCCTTGAGCTGC-3′. All plasmids were sequenced (Eurofins, Uppsala, Sweden) before use.

##### Antibodies

Mouse monoclonal anti-FLAG antibodies M2 (catalog no. F1804, lot no. SLB87188), and M5 (catalog no. F4042, lot no. 105k6067) were purchased from Sigma-Aldrich and used at 1:1,000 (v/v) dilution; the specificity of the anti-FLAG antibodies was verified based on the recognition of the appropriate transfected protein; the anti-FLAG antibody gave rise to some background bands that were easy to discriminate against. Rabbit polyclonal anti-PARP1 antibody (N-terminal) used for PLA with mouse anti-Smad1 (catalog no. 39559, lot no. 00909001) was purchased from Active Motif (La Hulpe, Belgium) and used at 1:1,080 (v/v) dilution; the specificity of the antibody was verified based on the electrophoretic mobility of the protein and its loss after siRNA-mediated silencing. Mouse monoclonal anti-PARP1 antibody used for immunoblotting (catalog no. 51-8114KC, lot no. 30826) was purchased from BD Pharmingen/Transduction Laboratories and used at 1:2,000 (v/v) dilution; the specificity of the antibody was verified based on the electrophoretic mobility of the protein and its loss after siRNA-mediated silencing. Mouse monoclonal anti-PARG antibody, clone D8B10 (catalog no. MABS61, lot no. 2490688), was purchased from Merck/Millipore and used at 1:1,000 (v/v) dilution; the specificity of the antibody was verified based on the electrophoretic mobility of the isoforms of this protein and their loss after siRNA-mediated silencing. Mouse monoclonal anti-Smad4 (B8) antibody (catalog no. sc-7966, lot no. E228) was purchased from Santa Cruz Inc. and used at 1:1,000 (v/v) dilution; the specificity of the antibody was verified based on the electrophoretic mobility of the protein, its loss after siRNA-mediated silencing, and its co-precipitation with R-Smads after TGFβ or BMP stimulation. Goat polyclonal anti-Smad6/7 (N-19) antibody (catalog no. sc-7004, lot no. J130) was purchased from Santa Cruz Inc. and used at 1:200 (v/v) dilution; the specificity of the antibody was verified based on the electrophoretic mobility of the protein compared with overexpressed Smad6 and Smad7 as molecular size marker and its loss after siRNA-mediated silencing. Rabbit polyclonal anti-ID1 (Z-8) antibody (catalog no. sc-428, lot no. B1306) was purchased from Santa Cruz Inc. and used at 1:200 (v/v) dilution; the specificity of the antibody was verified based on the electrophoretic mobility of the protein and its inducibility after BMP stimulation. Mouse monoclonal anti-β-actin antibody (catalog no. sc-69879, lot no. J1509) was purchased from Santa Cruz Inc. and used at 1:1,000 (v/v) dilution; the specificity of the antibody was verified based on the electrophoretic mobility of the protein appearing as single band. Mouse monoclonal anti-α-tubulin antibody (catalog no. sc-8035, lot no. E2909) was purchased from Santa Cruz Inc. and used at 1:1,000 (v/v) dilution; the specificity of the antibody was verified based on the electrophoretic mobility of the protein appearing as single band. Rabbit monoclonal anti-Smad1 antibody used for immunoblotting (catalog no. 1649-1, lot no. YD020503) was purchased from Epitomics (Burlingame, CA) and used at 1:1,000 (v/v) dilution; the specificity of the antibody was verified based on the electrophoretic mobility of the protein, its loss after siRNA-mediated silencing, and its co-precipitation with Smad4 after BMP stimulation. Mouse monoclonal anti-Smad1 antibody used for immunoprecipitation (catalog no. 913C1b, lot no. GR103403-5)was purchased from Abcam (Cambridge, UK) and used at 1:1,000 (v/v) dilution; the specificity of the antibody was verified based on the electrophoretic mobility of the protein, its loss after siRNA-mediated silencing, and its co-precipitation with Smad4 after BMP stimulation. Rabbit polyclonal anti-Smad1 antibody used for immunoblotting (catalog no. ab33902, lot no. GR179307-2) was purchased from Abcam (Cambridge, UK) and used at 1:1,000 (v/v) dilution; the specificity of the antibody was verified based on the electrophoretic mobility of the protein, its loss after siRNA-mediated silencing, and its co-precipitation with Smad4 after BMP stimulation. Rabbit monoclonal anti-phospho-Smad1(Ser^463/465^)/Smad5 (Ser^463/465^)/Smad8(Ser^426/428^) antibody (catalog no. 9511S, lot no. 8) was purchased from Cell Signaling Technology (Danvers, MA) and used at 1:1,000 (v/v) dilution; the specificity of the antibody was verified based on the electrophoretic mobility of the protein, its loss after siRNA-mediated silencing, and its inducibility after BMP stimulation. Rabbit polyclonal anti-Smad5 antibody (catalog no. 9517S, lot no. 4) was purchased from Cell Signaling Technology (Danvers, MA) and used at 1:1,000 (v/v) dilution; the specificity of the antibody was verified based on the electrophoretic mobility of the protein, its loss after siRNA-mediated silencing, and its co-precipitation with Smad4 after BMP stimulation. Mouse monoclonal anti-GAPDH antibody (catalog no. AM4300, lot no. 0711003) was purchased from Ambion (Life Technologies Corp., Foster City, CA) and used at 1:50,000 (v/v) dilution; the specificity of the antibody was verified based on the electrophoretic mobility of the protein appearing as single band. Rabbit polyclonal anti-phospho-Smad1(Ser^463/465^) antibody was produced in house as previously described (28), and its IgG content was purified providing a final concentration of 2 μg/ml; it was used at 1:5,000 (v/v) dilution; the specificity of the antibody was verified based on the electrophoretic mobility of the protein, its loss after siRNA-mediated silencing, and its inducibility after BMP stimulation. Mouse monoclonal anti-Myc (9E10) antibody was produced in house by culturing a mouse hybridoma cell line as previously described (30) and its IgG content was purified providing a final concentration of 2 μg/ml; it was used at 1:10,000 (v/v) dilution; the specificity was verified based on the recognition of the appropriate transfected protein; the anti-Myc antibody gave rise to few background bands that were easy to discriminate against. As secondary antibodies we used donkey anti-goat antibody conjugated to HRP (catalog no. sc-2020, lot no. H1715) purchased from Santa Cruz Inc. and used at 1:10,000 (v/v) dilution; goat anti-rabbit antibody conjugated to HRP (catalog no. 656120, lot no. 1576428A) purchased from Invitrogen/Life Technologies and used at 1:40,000 (v/v) dilution; and goat anti-mouse antibody conjugated to HRP (catalog no. 626520, lot no. 1629505A) purchased from Invitrogen/Life Technologies Corp. and used at 1:20,000 (v/v) dilution.

##### Immunoblotting

Total proteins from transfected and/or stimulated HaCaT or HEK293T cells were extracted in a Nonidet P-40 containing lysis buffer (20 mm Tris-HCl, pH 8.0, 1% Nonidet P-40, 150 mm NaCl, 2 mm EDTA, and complete protease inhibitor mixture from Roche). Then lysates were heated at 95 °C for 5 min and subjected to SDS-PAGE. The resolved proteins were transferred to nitrocellulose using a Bio-Rad wet or semidry transfer unit. The efficiency of immunoblotting and equal loading of proteins was verified by staining of the nictrocellulose membrane with 0.1% (w/v) Ponceau S in acetic acid. Upon incubation with primary antibodies and horseradish peroxidase-conjugated secondary antibodies (see list above), enhanced chemiluminescence assays were performed using the Millipore kit (Merck/Millipore), and the immunoblots were developed at ambient temperature and during the first few minutes of the reaction that corresponded to the linear range of light emission; immunoblots were exposed on x-ray films, which were scanned and quantified by AIDA software (FujiFilm Sweden, Stockholm, Sweden), or immunoblots were analyzed in an automated imaging system (Bio-Rad) and the corresponding software Quantity One. Digital image memory content was reduced, and brightness contrast was adjusted using Adobe Photoshop CS3.

##### Nucleocytoplasmic Fractionation

The nucleocytoplasmic fractionation was performed using the CelLytic NuCLEAR extraction kit from Sigma. Briefly, HaCaT cells were stimulated as described in the figure legend and collected by trypsinization. Hypotonic lysis buffer (10 mm HEPES, pH 7.9, 1.5 mm MgCl_2_, 10 mm KCl, and complete protease inhibitor mixture from Sigma-Aldrich) was added to the cells and incubated for 15 min on ice. Then cytoplasmic and nuclear fractions were isolated according to the protocol. For immunoprecipitations from nuclear fractions, the lysis buffer described above was used.

##### Co-immunoprecipitation Assays

HaCaT or HEK293T cells were transfected and/or stimulated as indicated, and total proteins were extracted in Nonidet P-40 lysis buffer, as described above. Then the proteins of interest were immunoprecipitated using specific antibodies. In case of endogenous co-immunoprecipitations, antibodies were precoupled to magnetic protein A/G Dynabeads (Invitrogen/Life Technologies) and incubated with the lysates overnight at 4 °C. For immunoprecipitation of transfected proteins, antibodies preconjugated to magnetic agarose beads (anti-mouse Dynabeads (M-280, catalog no. 11202D, lot no. 160461500) and anti-rabbit protein-A Dynabeads (catalog no. 10002D, lot no. 00315255) from Invitrogen/Life Technologies were used and incubated with lysates for 2 h at 4 °C. The immunocomplexes were washed four times in lysis buffer, resolved by SDS-PAGE, and immunoblotted with antibodies as described above.

##### GST Pulldown Assays

Plasmid DNA constructs encoding GST fusion proteins were transformed into the BL21 strain of *Escherichia coli* and BL21 expressing the GST fusion proteins were inoculated at 37 °C. Then proteins were extracted from bacteria using a Triton X-100 containing lysis buffer (50 mm Tris-HCl, pH 7.5, 1 mm EDTA, 100 mm NaCl, 5% glycerol, 0.5% Triton X-100), supplemented with 1 mm DTT and protease inhibitors, and incubated end over end at 4 °C, overnight, with glutathione-Sepharose beads (catalog no. 17-5132-01, lot no. 10172617; GE Healthcare). The next day the GST-proteins bound to beads were washed four times in GST pulldown buffer (50 mm Tris-HCl, pH 7.4, 100 mm NaCl, 1% Nonidet P-40, 2 mm MgCl_2_) and dissolved in GST pulldown buffer supplemented with protease inhibitors and sodium azide. Purified GST fusion proteins conjugated to glutathione beads were added to total cell lysates and incubated end over end at 4 °C for 2 h. The beads were then washed four times using Nonidet P-40 lysis buffer and before the last wash beads were transferred in new, clean tubes. Samples were subjected to SDS-PAGE, followed by immunoblotting as described above. Input of the GST fusion proteins were loaded on separate gels and subjected to staining by Coomassie Brilliant Blue and served as normalization controls.

##### In Vitro ADP-ribosylation Assays

Newly prepared GST or GST-Smad proteins were kept bound to glutathione beads and incubated in 100 μl of reaction buffer (100 mm Tris-HCl, pH 8, 10 mm MgCl_2_, 1 mm DTT), with or without 100 ng of PARP1 (Axxora, LLC/ENZO Life Sciences). Then 80 nm β-NAD^+^ and 20 nm ^32^P-β-NAD^+^ (PerkinElmer) were added, and the reactions were incubated for 30 min at 37 °C while shaking. At the end of each reaction, beads bound to GST fusion proteins were collected via centrifugation, followed by a quick double wash with ice-cold Nonidet P-40 lysis buffer to remove excess radioactive β-NAD^+^. Alternatively, a sample of the total reaction was collected. Samples were then heated for 4 min at 95 °C in sample buffer and loaded on gels. Gels were fixed, stained with Coomassie Brilliant Blue, and dried before measuring radioactivity in a Fuji-X Bio-Imager. The non-radioactive version of the same assay was performed exactly in the same manner, except that a total of 100 nm cold β-NAD^+^ was included in the reaction.

##### In Vitro De-ADP-ribosylation Assay

Freshly prepared GST and GST-Smads were immobilized on glutathione beads with 100 μl of reaction buffer (100 mm Tris-HCl, pH 8, 10 mm MgCl_2_, 1 mm DTT) along with 100 ng of PARP1 and increasing amounts of PARG (Axxora, LLC/ENZO Life Sciences). Then 80 nm β-NAD^+^ and 20 nm ^32^P-β-NAD^+^ were added to the mixture, and samples were incubated at 37 °C while shaking. After 30 min, beads were centrifuged at 4,000 rpm and washed three times with ice-cold Nonidet P-40 lysis buffer to remove unbound PARG, PARP1, and β-NAD^+^. Washed beads were then mixed with sample buffer with β-mercaptoethanol and heated for 4 min at 95 °C and loaded on gels. Gels were then fixed, stained with Coomassie Brilliant Blue, and dried to measure the radioactivity by a Fuji-X Bio-Imager.

##### Proximity Ligation Assay

HaCaT cells were washed with PBS twice for 5 min with agitation and then permeabilized with 0.2% Triton X-100 in PBS for 10 min at room temperature with agitation and washed with PBS again for 10 min. The cells were blocked by incubating with Duolink II blocking solution for 1 h at room temperature with agitation (80 rpm) and removed prior to adding primary antibodies. The antibodies were diluted in Duolink II antibody diluent 1:100, and the cells were incubated overnight at 4 °C. The cells were washed three times for 3 min with buffer A (Duolink; Olink Bioscience, Uppsala, Sweden) prior to incubating with secondary probes (Duolink II), diluted with Duolink II antibody diluent 1:5. The cells were further incubated for 2 h at 37 °C with agitation (80 rpm), prior to washing three times, for 3 min with Buffer A. Duolink ligation stock was diluted 1:5 in double-distilled water and Duolink ligase was added to the ligation solution from the previous step at a 1:40 dilution, and the mixture was vortexed. Ligation solution was added to each sample, and the slides were incubated in a preheated humidified chamber at 37 °C for 30 min. The slides were washed with buffer A twice for 2 min under gentle agitation. Duolink amplification stock was diluted 1:5 in double-distilled water, and amplification solution was added at 1:80 dilution while vortexing. Amplification solution was added to each sample and the slides were incubated in a preheated humidified chamber at 37 °C for 90 min and then washed once with Buffer A for 5 min at room temperature with gentle agitation. Phalloidin 488 (1:40) and Hoechst (1:500) (both purchased from Sigma-Aldrich) were added to PBS, and the slides were incubated at room temperature for 10 min prior to two washes for 10 min with buffer B (Duolink II). The slides were rinsed with double-distilled water and mounted with Slowfade (Invitrogen/Life Technologies-Thermo Fischer Scientific) mounting medium. Pictures were taken with a Zeiss LSM-510 inverted confocal microscope equipped with a Hamamatsu C4742-95 digital camera, using the Zeiss Plan-neofluar 20×/0.75 objective lens and photographing at ambient temperature without immersion oil. The DuolinkImageTool software (Olink Bioscience) was used for image analysis and signal quantification. Because of the antibody species specificity requirement in PLA assays, a rabbit anti-PARP1 antibody was combined with a mouse anti-Smad1 antibody (see Fig. 7, *D* and *E*).

##### Real Time RT-PCR

Total RNA from HaCaT or HEK293T cells was extracted using the RNeasy kit (Qiagen) according to the protocol of the manufacturer. cDNA was synthesized using the iScript cDNA synthesis kit from Bio-Rad. Real-time PCR was done using iTaq SYBR green supermix with ROX from Bio-Rad in triplicate. Controls without reverse transcriptase (−RT) or without cDNA (water) were also included in every quantitative PCR assay. Gene expression levels were determined by the comparative Ct method and using GAPDH or HPRT1 (hypoxanthine phosphor-ribosyl transferase 1) as reference. Normalized mRNA expression levels are plotted in bar graphs that represent average values from triplicate determinations with standard deviations. Each independent experiment was repeated at least three times. The primers used for quantitative PCR amplification were: human *PARP1* forward, 5′-AAGCCCTAAAGGCTCAGAACG-3′, reverse, 5′-ACCATGCCATCAGCTACTCGGT-3′; human *PARG* forward, 5′-GAAAGGGACGACTGGCAGCGG-3′, reverse, 5′-CCAAAGGCACCACAGCCCCA-3′; human *GAPDH* forward, 5′-GGAGTCAACGGATTTGGTCGTA-3′, reverse, 5′-GGCAACAATATCCACTTTACCA-3′; human *SMAD6* forward, 5′-GGCTCTGCGGGCCCGAATC-3′, reverse, 5′-GAGACATGCTGGCGTCTGAGAA-3′; human *SMAD7* forward, 5′-ACCCGATGGATTTTCTCAAACC-3′, reverse, 5′-GCCAGATAATTCGTTCCCCCT-3′; human *ID1* forward, 5′-GGACGAGCAGCAGGTAAACG-3′, reverse, 5′-TGCTCACCTTGCGGTTCTG-3′; and human *HPRT1* forward, 5′-GCTTCCTCCTCCTGAGCAGTC-3′, reverse, 5′-CACTAATCACGACGCCAGGGCTGC-3′.

##### Luciferase Assay

C2C12 cells stably transfected with the BMP/Smad-responsive promoter reporter construct (BRE_2_-luc) and PARP1^−/−^ MEFs were transfected with siRNAs or plasmids prior to BMP7 stimulation for 7–18 h as indicated in the figure legends. The activity of β-galactosidase, derived from a plasmid that was also stably transfected into C2C12 cells was measured for normalization of the measurements. All cells were lysed in lysis buffer containing 5 mm Tris-phosphate buffer, pH 7.8, 2 mm DTT, 2 mm trans-1,2-diaminocyclohexane-*N*,*N*,*N′*,*N′*-tetra-acetic acid, 5% glycerol, and 1% Triton X-100. The β-galactosidase assay was performed by mixing the cell lysate with 100 mm sodium phosphate, pH 7.3, 1 mm MgCl_2_, 50 mm β-mercaptoethanol, and 0.67 mg/ml of *o*-nitrophenyl β-d-galactopyranoside, and the absorbance was monitored at 420 nm. Luciferase reporter assays were performed with the enhanced firefly luciferase assay kit from either BD PharMingen, Inc. (Life Technologies) or from Biotium Inc. (Hayward, CA), according to the manufacturers' protocol. Normalized promoter activity data are plotted in bar graphs that represent average values from triplicate determinations with standard deviations. Each independent experiment was repeated at least twice.

##### Alkaline Phosphatase Assay

C2C12 mouse myoblasts were treated with BMP7 and/or transfected with siRNAs or plasmid constructs as explained in the figures. The cells were washed two times with PBS and then lysed in Triton X-100 containing ALP buffer (1 mm MgCl_2_, 0.1 mm ZnCl_2_, 100 mm glycine, 0.1% Triton X-100, pH 10.5). After the addition of ALP lysis buffer, cells were incubated for 1 h on ice, on an orbital shaker; then lysates were collected and centrifuged at 5,000 rpm for 4 min. Then supernatant was collected and used for the reaction with the alkaline phosphatase substrate *p*-nitrophenyl phosphate. Samples with substrate were incubated at 37 °C for 30 min to 3 h until a yellow soluble pigment was produced. Triplicates were used per each condition, and then absorbance was measured at 405 nm. Measurements were normalized to the total amount of protein in each sample, measured with the bicinchoninic acid protein assay kit (Pierce, Thermo Fischer Scientific) according to the manufacturer's protocol.

##### Statistical Analysis

The differences between mRNA levels or reporter luciferase activity under control, gene specific silencing, and protein overexpression conditions, were evaluated statistically using a standard two-tailed *t* test for samples with unequal variance and two-sample with equal variance, respectively. Significance is reported at *p* < 0.05 or at *p* < 0.01. The data are plotted in bar graphs that represent average values from triplicate determinations with standard deviations. Each experiment was repeated two to five times, which represents biological repeats, and each of these included three technical repeats.

## Results

### 

#### 

##### The PARG Glycohydrolase Has a Positive Contribution to BMP Signaling

**FIGURE 1.**
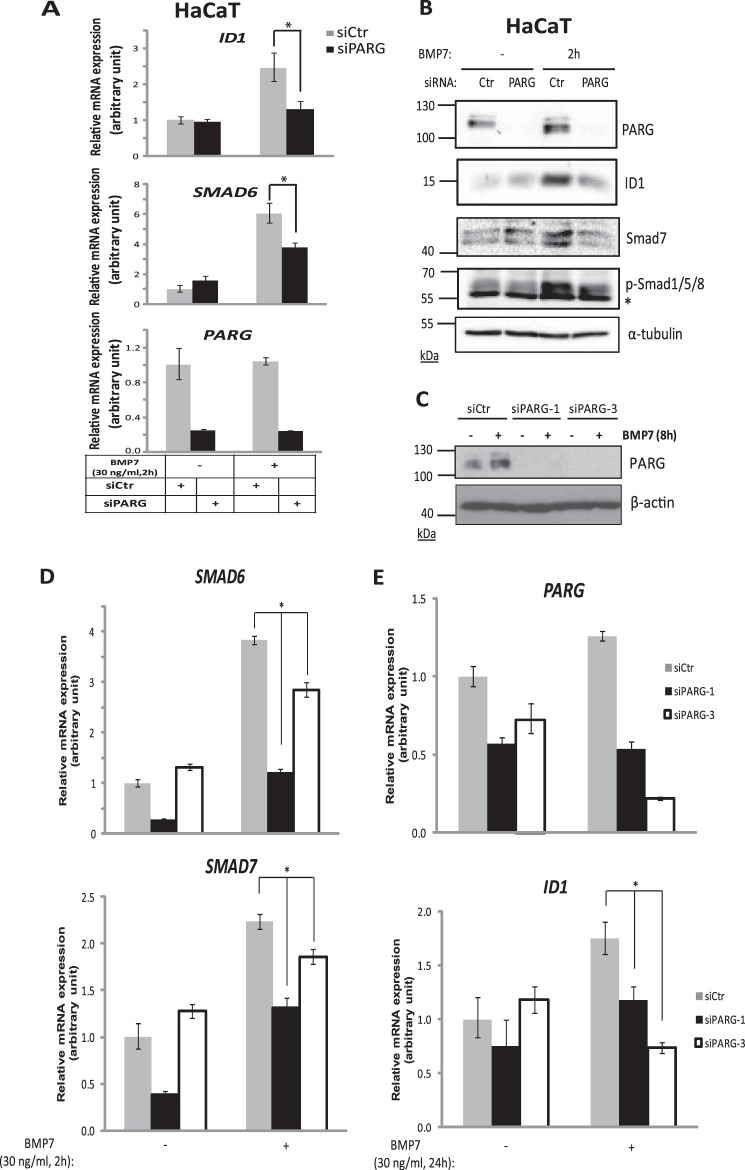
**Depletion of PARG inhibits BMP target gene expression.** *A*, transient silencing of PARG in HaCaT cells, using specific siRNA targeting PARG or non-targeting siRNA (*siCtr*) and in the presence or absence of 30 ng/ml BMP7 stimulation for 2 h, followed by qRT-PCR for *ID1*, *SMAD6*, and *PARG* mRNAs. The *bars* represent the relative mRNA expression of the corresponding genes, normalized to the expression of the housekeeping gene *GAPDH*. Mean values from triplicate determinations are shown in the graph, and standard deviation is presented with *error bars. Asterisks* indicate a statistically significant difference at *p* < 0.05. *B*, immunoblotting of lysates from HaCaT cells, transiently transfected with siRNA targeting PARG or non-targeting siRNA (*Ctr*), with or without stimulation of 30 ng/ml BMP7 for 2 h. Specific antibodies for PARG, ID1, Smad7, phospho-Smad1/5/8, and α-tubulin were used for the detection of the corresponding protein levels. α-Tubulin serves as a loading control, and phospho-Smad1/5/8 was used to evaluate the efficiency of BMP7 stimulation. An *asterisk* indicates a nonspecific protein band. A representative immunoblot of four repeats is shown. Molecular size markers in kDa are also marked. *C*, immunoblotting of lysates from HaCaT cells, transiently transfected with two individual siRNAs targeting PARG (siPARG-1 and siPARG-3) or non-targeting siRNA (*siCtr*), with or without stimulation of 30 ng/ml BMP7 for 8 h. A specific antibody was used for the detection of PARG protein levels. β-Actin serves as a loading control. A representative immunoblot of three repeats is shown. Molecular size markers in kDa are also marked. *D*, transient silencing of PARG in HaCaT cells, using two individual siRNAs targeting *PARG* (siPARG-1 and siPARG-3) or non-targeting siRNA (*siCtr*) and in the presence or absence of 30 ng/ml BMP7 stimulation for 2 h, followed by qRT-PCR for *SMAD6* and *SMAD7* mRNAs. The *bars* represent the relative mRNA expression of the corresponding genes, normalized to the expression of the housekeeping gene *HPRT1*. The statistical analysis was performed as explained in *A. E*, transient silencing of PARG in HaCaT cells, using two individual siRNAs targeting *PARG* (siPARG-1 and siPARG-3) or non-targeting siRNA (*siCtr*) and in the presence or absence of 30 ng/ml BMP7 stimulation for 24 h, followed by qRT-PCR for *PARG* and *ID1* mRNAs. The data are presented as in *A* and *D*.

**FIGURE 2.**
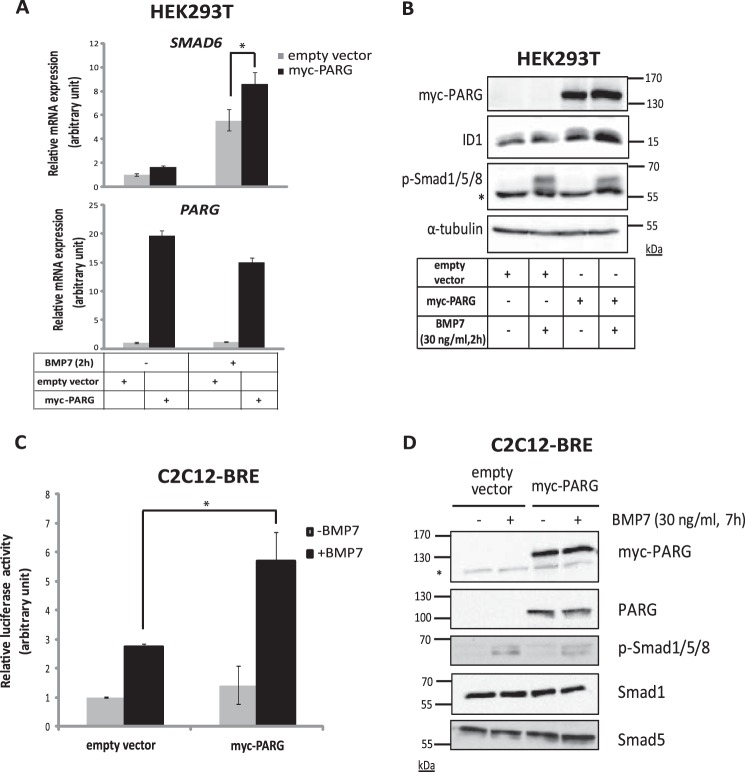
**PARG positively contributes to BMP signaling.** *A*, transient overexpression of PARG in HEK293T cells, using myc-PARG or the empty vector (pCS2) plasmid DNA, in the absence or presence of 30 ng/ml BMP7 stimulation for 2 h, followed by qRT-PCR for *SMAD6* and *PARG* mRNAs. The data are presented as in Fig. 1*A*. An *asterisk* indicates a statistically significant difference at *p* < 0.05. *B*, immunoblotting of lysates from HEK293T cells from the experiment shown in *A*, transiently transfected with myc-PARG or the empty vector (pCS2) plasmid DNA and after stimulation with 30 ng/ml BMP7 or vehicle control for 2 h. Specific antibodies for the detection of PARG, ID-1, and phospho-Smad1/5/8 protein (*asterisk* indicates a nonspecific protein band) levels and α-tubulin, which was used as a loading control for the assay. A representative immunoblot of three repeats is shown. Molecular size markers in kDa are also marked. *C*, luciferase reporter assay in C2C12 BRE-luc cells transiently transfected with myc-PARG or the control empty vector (pCS2) constructs without or with stimulation of cells with 30 ng/ml BMP7 for 7 h. Mean values from triplicate determinations normalized to β-galactosidase measurements along with the corresponding standard deviation are shown in the graph. An *asterisk* indicates a statistically significant difference at *p* < 0.05. *D*, immunoblotting of cell lysates from the experiment shown in *C* as a control for evaluating the transfection efficiency of the indicated plasmids. Immunoblotting was performed using specific antibodies for myc-PARG (anti-myc tag antibody), PARG, and phospho-Smad1/5/8 to measure the efficiency of BMP7 stimulation and total Smad1 and Smad5 protein levels. An *asterisk* indicates nonspecific protein bands. A representative immunoblot of three repeats is shown. Molecular size markers in kDa are also marked.

Influenced from the strong positive impact of PARG on TGF-β signaling (23), we examined the role of endogenous PARG on BMP signaling in various established cell models. Silencing of endogenous PARG in normal human HaCaT keratinocytes, using a pool of four PARG-specific siRNAs significantly reduced the induction of *ID1* and *Smad6* by BMP7 (Fig. 1*A*). These are two well established direct target genes of BMP signaling in diverse cell types (9–11). The same results were observed by analyzing ID1 and Smad7 protein expression after BMP7 stimulation and PARG silencing in HaCaT cells (Fig. 1*B*). Repeating these experiments with two additional, individual siRNAs, siPARG-1 and siPARG-3, that potently silenced the endogenous HaCaT PARG protein (Fig. 1*C*) led to significant reduction of *Smad6*, *Smad7*, and *ID1* induction by BMP7 (Fig. 1, *D* and *E*). PARG overexpression in HEK293T cells conversely enhanced *Smad6* mRNA (Fig. 2*A*) and ID1 protein induction (Fig. 2*B*) by BMP7. Attempts to perform the PARG overexpression in the more physiological cell model of HaCaT cells did not succeed because of low expression efficiency of the PARG vector (unpublished results).

To assess a more general impact of PARG on BMP signaling mediated by Smad proteins, we employed the mouse C2C12 pluripotent cell model that differentiates toward the osteoblastic lineage upon stimulation with BMPs, including BMP7 (8). To analyze the functional consequence of PARG overexpression on direct BMP-Smad transcriptional activity, we performed transcriptional signaling assays in stably transfected cells expressing a Smad1/5-specific promoter-reporter construct, BRE_2_-luc, derived from the *ID1* gene (10). Transfection of PARG in the C2C12-BRE-luc cell model significantly enhanced the BRE_2_-luc transcriptional activity in response to BMP7 (Fig. 2, *C* and *D*). Analysis of receptor-phosphorylated and total protein levels of Smad1 and Smad5 did not reveal any significant changes after PARG overexpression, suggesting that PARG acted by modulating the transcriptional activity of Smads (Fig. 2*D*). All these experiments convincingly showed that PARG is a positive regulator of BMP-mediated transcriptional responses controlling different target genes of the BMP signaling pathway.

##### PARG Interacts with Smad5

**FIGURE 3.**
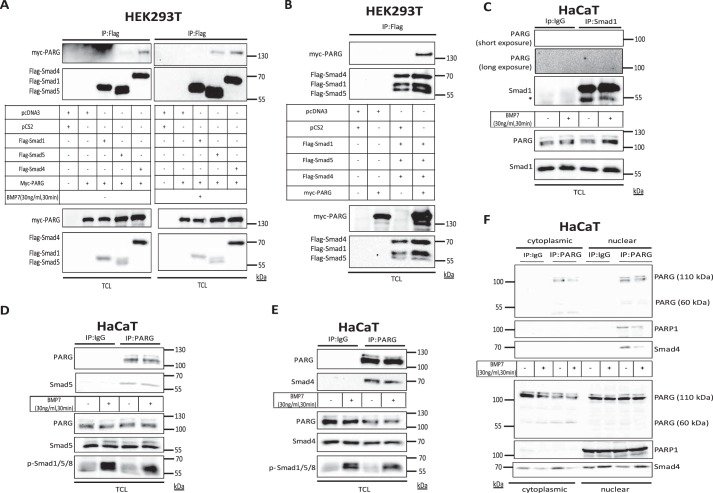
**PARG forms complexes with Smad5 and Smad4.** *A*, immunoprecipitation (*IP*) of FLAG-Smad1, FLAG-Smad5, or FLAG-Smad4, followed by immunoblotting for myc-PARG in transiently transfected HEK293T cells with the indicated plasmids without (*left panel*) or with stimulation with 30 ng/ml BMP7 for 30 min (*right panel*). Immunoblots from total cell lysates (*TCL*) show the expression levels of the transfected proteins. A representative immunoblot of three repeats is shown. Molecular size markers in kDa are also marked. *B*, immunoprecipitation of FLAG-Smad1/4/5 followed by immunoblot for myc-PARG in transiently transfected HEK293T cells with the indicated plasmids. The expression levels of transfected proteins are presented in the corresponding immunoblots from TCL. A representative immunoblot of three repeats is shown. Molecular size markers in kDa are also marked. *C*, endogenous immunoprecipitation of Smad1 followed by immunoblotting for endogenous PARG and Smad1 in cell lysates from HaCaT cells, in the absence or presence of stimulation with 30 ng/ml BMP7 for 30 min. Immunoprecipitation with nonspecific IgG served as a negative control. An *asterisk* shows the heavy Ig chain. Expression levels of endogenous proteins are shown in TCL immunoblots. Because of the absence of PARG signal in the co-immunoprecipitation, two different exposures are shown to clarify the identity of the void immunoblot. A representative immunoblot of three repeats is shown. Molecular size markers in kDa are also marked. *D*, immunoprecipitation of endogenous PARG followed by immunoblotting for endogenous Smad5 and PARG in cell lysates from HaCaT cells, in the absence or presence of stimulation with 30 ng/ml BMP7 for 30 min. Precipitation with nonspecific IgG served as a negative control. Expression levels of endogenous PARG and Smad5 proteins are shown in TCL immunoblots. Phospho-Smad1/5/8 (*p-Smad1/5/8*) levels serve as control to evaluate the BMP7 stimulation. A representative immunoblot of three repeats is shown. Molecular size markers in kDa are also marked. *E*, immunoprecipitation of endogenous PARG followed by immunoblotting for endogenous Smad4 and PARG in cell lysates from HaCaT cells, in the absence or presence of stimulation with 30 ng/ml BMP7 for 30 min. Immunoprecipitation with nonspecific IgG served as a negative control. Expression levels of endogenous PARG, Smad4, and phospho-Smad1/5/8 proteins are shown in TCL immunoblots. A representative immunoblot of three repeats is shown. Molecular size markers in kDa are also marked. *F*, immunoprecipitation of endogenous PARG, followed by immunoblotting for endogenous Smad4, PARP1, and PARG from cytoplasmic or nuclear HaCaT extracts. The cells were stimulated with 30 ng/ml BMP7 for 30 min or left untreated, and immunoprecipitation with a nonspecific IgG was used as a negative control. Expression levels of endogenous PARG, Smad4, and PARP1 in cytoplasmic or nuclear cell extracts are also shown. A representative immunoblot of three repeats is shown. Molecular size markers in kDa are also marked.

To start exploring how PARG can regulate BMP signaling, we tested whether PARG could form complexes with Smad proteins of the BMP pathway, including Smad1, Smad5, and Smad4. Co-immunoprecipitation experiments examined the association of Smad1, Smad5, and Smad4 (as positive control) with PARG (Fig. 3*A*). Co-transfection of HEK293T cells with Smad1, Smad5, and Smad4 revealed a complex of PARG with Smad5 and Smad4, whereas Smad1 did not show interaction with PARG (Fig. 3*A*). Brief (30 min) stimulation with BMP7 significantly enhanced the complexes between PARG and Smad5 or Smad4, whereas a Smad1-PARG complex remained undetectable (Fig. 3*A*). In cells co-transfected with all three Smads—Smad1, Smad5, and Smad4—their complex with PARG was much easier to observe (Fig. 3*B*). These results were confirmed at the endogenous level using the HaCaT keratinocyte model (Fig. 3, *C–E*). Endogenous Smad1 failed to form detectable complexes with endogenous PARG (Fig. 3*C*). Endogenous Smad5 formed complexes that were not further enhanced by BMP7 stimulation (Fig. 3*D*). Endogenous Smad4 (positive control) also formed complexes with endogenous PARG, and BMP7 stimulation could not reveal any further enhancement of the association (Fig. 3*E*). To identify in which subcellular compartment the PARG-Smad4 complexes are formed, we performed endogenous immunoprecipitation between PARG and Smad4 in nuclear and cytoplasmic HaCaT cell lysates. The PARG-Smad4 complexes were observed only in the nuclear fraction. In addition, PARG also interacted with PARP1 exclusively in the nucleus (Fig. 3*F*). Thus, PARG can associate with BMP pathway Smads. However, PARG seems to form more stable complexes with Smad5 compared with Smad1.

##### PARP1 Opposes the Function of PARG in Mediating BMP-dependent Gene Responses

**FIGURE 4.**
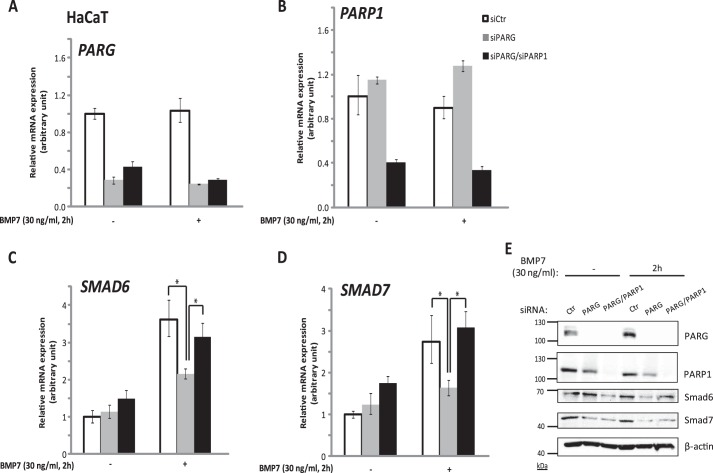
**PARP1 opposes the function of PARG during regulation of BMP target gene expression.** *A–D*, transient silencing of (i) PARG; (ii) PARG and PARP1 simultaneously in HaCaT cells, using specific siRNAs targeting PARG or PARP1; or (iii) non-targeting siRNA (*siCtr*) and left unstimulated or stimulated with 30 ng/ml BMP7 for 2 h, followed by qRT-PCR for *PARG* (*A*), *PARP1* (*B*), *SMAD6* (*C*), and *SMAD7* (*D*) mRNAs. Each siRNA for the double transfections was used at a concentration of 20 nm (total concentration, 40 nm), and in the condition where only PARG was silenced, the total siRNA concentration was adjusted by using 20 nm of siCtr siRNA. The data are presented as in Fig. 1*A. Asterisks* indicate a statistically significant difference at *p* < 0.05. *E*, immunoblotting of lysates from HaCaT cells, transiently transfected with siRNA targeting (i) PARG, (ii) *PARG* and *PARP1* simultaneously, or (iii) non-targeting siRNA (*Ctr*), with or without stimulation of 30 ng/ml BMP7 for 2 h. Specific antibodies for PARG, PARP1, Smad6, Smad7, and β-actin were used for the detection of the corresponding protein levels. β-Actin served as a loading control. A representative immunoblot of three repeats is shown. Molecular size markers in kDa are also marked.

Because PARG acts on proteins that are ADP-ribosylated and deribosylates them (18), we anticipated that the positive effects of PARG on BMP signaling would be counteracted by an ADP-ribosylating enzyme. We focused on PARP1 because we previously established that PARP1 ADP-ribosylates and negatively regulates Smads of the TGF-β signaling pathway (22). We therefore silenced both PARG and PARP1 simultaneously (Fig. 4, *A* and *B*) and analyzed target gene expression after BMP7 stimulation (Fig. 4, *C–E*). Although single PARG knockdown significantly reduced the inducible expression of *Smad6* and *Smad7* by BMP7 stimulation, knockdown of both PARG and PARP1 normalized the inducible expression of these two genes (Fig. 4, *C* and *D*), suggesting that PARG had an impact on ADP-ribosylation mediated by PARP1 and not by other, independent ADP-ribosylating enzymes. Similar results were obtained by studying expression of Smad6 and Smad7 proteins (Fig. 4*E*). Available antibodies make the analysis of endogenous Smad6 and Smad7 proteins very difficult because their levels are very low, and for this reason, the BMP7-inducible levels of these two proteins appeared only slightly higher than control; however, PARG knockdown reproducibly reduced Smad6 and Smad7 levels after BMP7 stimulation, whereas PARG/PARP1 double knockdown caused a relative (but not complete) normalization of the protein levels after BMP7 stimulation (Fig. 4*E*). The effects of the siRNAs on basal levels of Smad6 and Smad7 were less significant, suggesting that PARG and PARP1 modulate the inducible expression of these genes upon BMP signaling. These experiments demonstrated that PARG and PARP1 regulate BMP signaling co-dependently and not by acting through independent, parallel mechanisms.

##### PARP1 Inhibits Transcriptional Signaling by BMP

**FIGURE 5.**
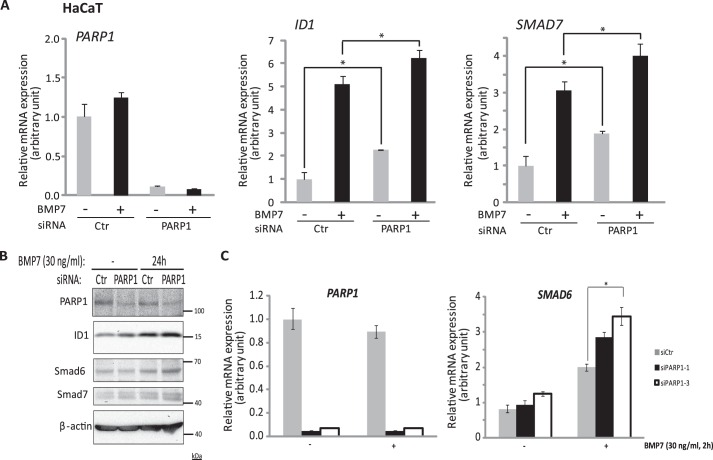
**Silencing of PARP1 enhances BMP-induced gene expression.** *A*, transient silencing of PARP1, using specific siRNA targeting *PARP1* or non-targeting siRNA (*Ctr*) as a negative control in HaCaT cells, and in the presence or absence of 5 ng/ml BMP7 stimulation for 2 h, followed by qRT-PCR for *PARP1*, *ID1*, and *SMAD7* mRNAs. The data are presented as in Fig. 1*A. Asterisks* indicate a statistically significant difference at *p* < 0.05. *B*, immunoblotting of lysates from HaCaT cells, transiently transfected with siRNA targeting *PARP1* or non-targeting siRNA (*Ctr*), with or without stimulation of 30 ng/ml BMP7 for 24 h. Specific antibodies for PARP1, ID1, Smad6, Smad7, and β-actin were used for the detection of the corresponding protein levels. β-Actin served as a loading control. A representative immunoblot of three repeats is shown. Molecular size markers in kDa are also marked. *C*, transient silencing of PARP1, using two individual siRNAs targeting *PARP1* (siPARP1-1 and siPARP1-3) or non-targeting siRNA (*Ctr*) as negative control in HaCaT cells and in the presence or absence of 30 ng/ml BMP7 stimulation for 2 h, followed by qRT-PCR for *PARP1* and *SMAD6* mRNAs. The data are presented as in *A. Asterisks* indicate a statistically significant difference at *p* < 0.05.

**FIGURE 6.**
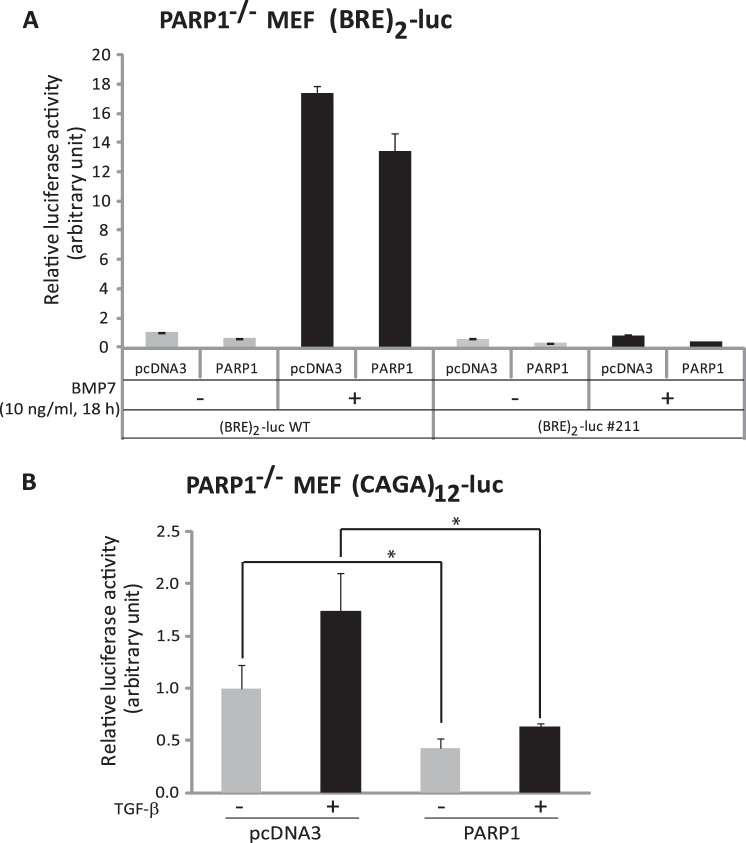
**PARP1 down-regulates BMP signaling.** *A*, luciferase reporter assay in PARP1^−/−^ MEFs transiently transfected with myc-PARP1 or control empty vector (pcDNA3) plasmid DNA and in the absence or presence of 10 ng/ml BMP7 for 18 h. The non-stimulated cells were treated with 0.5 μm dorsomorphin homolog 1, and the BMP7-stimulated cells were treated with DMSO. A wild-type BRE_2_-luciferase reporter (BRE_2_-luc WT) or a mutant BRE_2_-luciferase reporter (BRE_2_-luc #211) and the β-galactosidase constructs were also transiently co-transfected in these cells. The data are presented as in Fig. 2*C. B*, luciferase reporter assay in PARP1^−/−^ MEFs transiently transfected with myc-PARP1 or a control empty vector (pcDNA3) plasmid DNA with or without addition of 1 ng/ml TGF-β1 for 18 h. The Smad-binding element or CAGA_12_-luciferase reporter construct (CAGA_12_-luc) and the β-galactosidase constructs were also transiently co-transfected into the cells. The data are presented as in Fig. 2*C. Asterisks* indicate a statistically significant difference at *p* < 0.05.

The previous experiment suggested that similar to the TGF-β signaling pathway (22), PARP1 might negatively regulate BMP signaling. To test this possibility more rigorously, we performed gene expression and transcriptional signaling assays after single PARP1 interference, using a pool of four individual siRNAs. Performing the knockdown of endogenous PARP1 in HaCaT keratinocytes followed by stimulation of the cells with BMP7 and analysis of endogenous mRNA expression showed that BMP-induced *ID1* and *Smad7* were further enhanced after PARP1 silencing (Fig. 5*A*). Similar results were obtained when ID1, Smad6 and Smad7 proteins were analyzed in HaCaT cells stimulated with BMP7 (Fig. 5*B*). Silencing PARP1 enhanced both basal and even more markedly the BMP7-inducible levels of the three proteins that record the signaling activity of the BMP pathway (Fig. 5*B*). Repeating these experiments with two additional, individual siRNAs, siPARP1-1 and siPARP1-3, which potently silenced endogenous HaCaT *PARP1* mRNA (Fig. 5*C*) and protein,^5^ led to significant enhancement of Smad6 induction by BMP7 (Fig. 5*C*). The potency of the individual siRNAs was not as good as the potency of the pool of four siRNAs (Fig. 5, compare *A* with *C*). To strengthen this evidence, we employed MEFs from the PARP1 knock-out mouse (PARP1^−/−^ MEFs (29)) and reconstituted wild-type PARP1 together with the Smad1/5-specific *ID1* promoter-reporter construct, BRE_2_-luc (10). Reconstitution of wild-type PARP1 was sufficient to inhibit the BMP-specific reporter under both basal and BMP7-stimulated conditions (Fig. 6*A*). As a negative control, we analyzed a mutant BRE_2_-luc promoter-reporter (Fig. 6*A*, BRE_2_-luc#211) that carries mutations in many (but not all) of the Smad-binding elements of the promoter, and as a result it shows weak inducibility of the reporter gene by BMP signaling (10). Reconstitution of PARP1 into the knock-out MEFs showed only marginal effects on reporter activity (Fig. 6*A*). PARP1 neither enhanced nor reduced significantly the activity of this promoter-reporter, as expected, because the promoter was only weakly induced (1.7-fold) by BMP7, compared with the 18-fold inducibility of the wild-type promoter (Fig. 6*A*). As a positive control we also analyzed a TGF-β-specific reporter, CAGA_12_-luc that is activated by Smad3 and Smad4 and found that this reporter was potently inhibited by wild-type PARP1 reconstitution in the knock-out cells (Fig. 6*B*). Thus, PARP1 acts as a negative regulator of BMP signaling at the level of Smad transcriptional function.

##### PARP1 Interacts with Smad1 and Smad5

**FIGURE 7.**
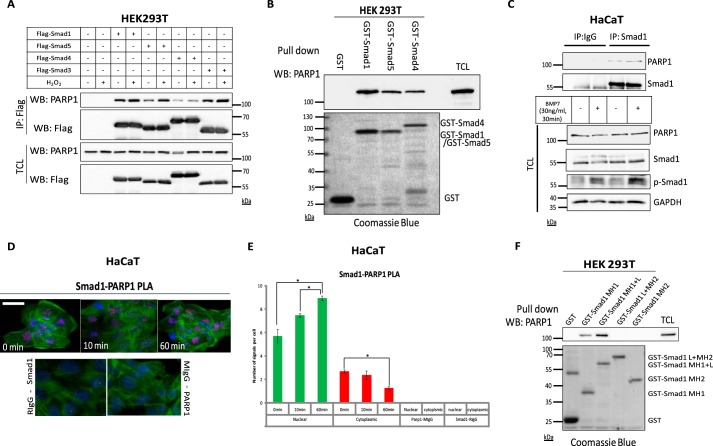
**PARP1 interacts with Smad1 and Smad5.** *A*, immunoprecipitation (*IP*) of FLAG-Smad1, FLAG-Smad5, FLAG-Smad3, or FLAG-Smad4, followed by immunoblotting for endogenous PARP1 in transiently transfected HEK293T cells with the indicated plasmids and in the absence or presence of 10 mm H_2_O_2_ for 10 min, which induces PARP1 activity. Immunoblots from total cell lysates (TCL) show the expression levels of the corresponding transfected and endogenous proteins. A representative immunoblot of three repeats is shown. Molecular size markers in kDa are also marked. *B*, pulldown assay with GST, GST-Smad1, GST-Smad5, and GST-Smad4 semipurified from *E. coli* and endogenous PARP1 from HEK293T cells detected by immunoblotting; Coomassie Brilliant Blue staining of the gel illustrates the amounts and quality of recombinant proteins. TCL is also immunoblotted as a marker of the expression level of endogenous PARP1. A representative immunoblot of three repeated pulldown assays is shown. Molecular size markers in kDa are also marked. *C*, immunoprecipitation of endogenous Smad1 followed by immunoblotting with endogenous PARP1, Smad1, and p-Smad1 in HaCaT cells stimulated with vehicle or BMP7 (30 ng/ml) for 30 min. IgG precipitation is shown as negative control. TCL shows the total level of endogenous proteins, p-Smad1, which serves as control for BMP stimulation and GAPDH, which serves as protein loading control. A representative immunoblot of three repeats is shown. *D*, PLA in HaCaT cells without or with stimulation with 30 ng/ml BMP7 for the indicated time periods, using specific Smad1 and PARP1 antibodies. Specific RCA signals were detected in the nuclei and cytoplasm (significantly less compared with nuclear signals). PLA using a specific PARP1 antibody with a nonspecific mouse IgG (MIgG) antibody or a specific Smad1 antibody in combination with a nonspecific rabbit IgG (RIgG) antibody served as negative controls for the specificity of the PLA assays. Signals from PLA are presented in *red*, DAPI staining for nuclei is in *blue*, and phalloidin for actin cytoskeleton staining is in *green*. A representative set of photomicrographs of three repeats is shown. A *bar* indicates 10 μm. *E*, quantification of the results from the experiment shown in *D* using the DuolinkImage Tool, with data for respective time points plotted as a histogram according to the number of RCA signals per cell, divided in cytoplasmic (*green bars*) and nuclear (*red bars*) signals. The figure shows a representative experiment from three repeats. *Asterisks* indicate a statistically significant difference at *p* < 0.05. *F*, pulldown assay with GST-fused Smad1 domains semipurified from *E. coli* and endogenous PARP1 from HEK293T cells detected by immunoblotting; Coomassie Brilliant Blue staining of the gel illustrates the amounts and quality of recombinant proteins. TCL is also immunoblotted as a marker of the expression level of endogenous PARP1. A representative immunoblot of three repeated pulldown assays is shown. Molecular size markers in kDa are also marked. *WB*, Western blotting.

To explore further how PARP1 can negatively regulate BMP signaling, we tested whether PARP1 could form complexes with Smad proteins of the BMP pathway. N-terminally FLAG-tagged Smad1, Smad3, Smad4, and Smad5 (Smad3 and Smad4 serving as positive controls) were transfected in HEK293T cells, and the formation of complexes with endogenous PARP1 was analyzed by co-immunoprecipitation assays (Fig. 7*A*). All four Smads formed readily detectable and reproducible complexes with endogenous PARP1. Note that in these experiments no stimulation of cells with BMP ligand was performed, suggesting that the transfected proteins acquire features of activated signaling simply because of the transfection condition. Indeed, a much stronger complex was detected when the cells were co-transfected with either Smad1 and Smad4 or Smad5 and Smad4.^5^ This implies that the association of Smad1 or Smad5 with PARP1 may require activation by the signaling pathway. As an additional positive control, we treated cells with peroxide, based on previous findings showing that brief activation of PARP1 by peroxide treatment could lead to complexes between PARP1 and Smad proteins of the TGF-β pathway (22). Indeed, both Smad1 and Smad5 formed complexes with PARP1 more efficiently after peroxide treatment, as did the positive control proteins, Smad3 and Smad4 (Fig. 7*A*).

We then used Smad1, Smad5, and Smad4 in fusion form with GST semipurified from *E. coli* lysates on glutathione-Sepharose and HEK293T cell extracts enriched in endogenous levels of PARP1 (Fig. 7*B*). We found that PARP1 formed complexes with Smad1 and Smad5 that were as strong as the complex with Smad4, which served as positive control, whereas no complex was formed with GST protein alone (Fig. 7*B*).

In agreement with the results of transfected or bacterially expressed Smads, endogenous Smad1 from human HaCaT keratinocytes co-immunoprecipitated with endogenous PARP1,and the complex was further enhanced after a 30-min stimulation of the cells with BMP7 (Fig. 7*C*). To analyze more carefully the time-dependent formation of endogenous complexes between PARP1 and Smad1, we performed PLA in HaCaT cells using antibodies recognizing the specific endogenous proteins (Fig. 7*D*). Complexes were recorded at all time points of the time course experiment; however, the number of endogenous complexes significantly rose after 10 min of stimulation with BMP7 (Fig. 7, *D* and *E*). The detected protein complexes resided mainly in the nuclei of the cells, but cytoplasmic complexes were also recorded (Fig. 7*E*). Interestingly, upon BMP7 stimulation, the number of cytoplasmic complexes decreased as the number of nuclear complexes increased (Fig. 7*E*).

Finally, using a panel of four deletion mutants of Smad1 fused to GST and similar to above HEK293T extracts enriched in endogenous PARP1 revealed that PARP1 associated with the N-terminal MH1 domain of Smad1, whereas the C-terminal MH2 domain failed to exhibit detectable association (Fig. 7*F*). We therefore conclude that PARP1 and Smad1 (and also Smad5) interact with each other at the endogenous level, their interaction can be enhanced by brief stimulation with BMP ligand or peroxide, and the N-terminal MH1 domain of the Smad protein seems to be responsible for the interaction.

##### PARP1 ADP-ribosylates and PARG Deribosylates BMP Smads

**FIGURE 8.**
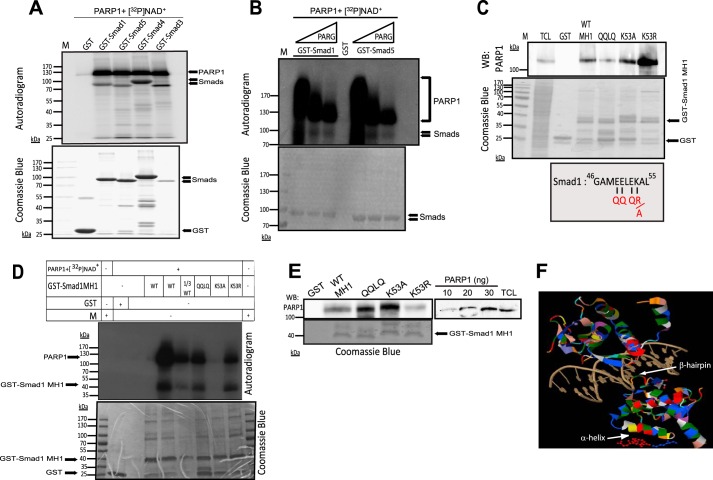
**PARP1 ADP-ribosylates, whereas PARG de-ADP-ribosylates Smad1 and Smad5.** *A*, *in vitro* ADP-ribosylation assay of Smad1, Smad5, Smad4, and Smad3. GST-Smad proteins were incubated with ^32^P-β-NAD^+^ and recombinant PARP1. After glutathione-agarose pulldown, ADP-ribosylated GST-Smad1/5/4/3 were imaged by autoradiography. The radioactive protein bands of PARP1 and GST-Smads are marked. The *lower panel* shows GST-Smad proteins stained with Coomassie Brilliant Blue after SDS-PAGE. *M*, molecular size marker. A representative autoradiogram of four assays is shown. Molecular size markers in kDa are also marked. *B*, *in vitro* de-PARylation of GST-Smad1 and GST-Smad5. PARG or vehicle were incubated with equal amounts of GST-Smad1/5, ^32^P-β-NAD^+^, and recombinant PARP1 for 30 min at 37 °C. ADP-ribosylated proteins were imaged by autoradiography. The radioactive protein bands of PARP1 and GST-Smads are marked. The *lower panel* shows total GST proteins stained with Coomassie Brilliant Blue. *M*, molecular size marker. A representative autoradiogram of five assays is shown. Molecular size markers in kDa are also marked. *C*, immunoblot of endogenous PARP1 from HEK293T cell extracts bound to the indicated GST-Smad1 MH1 domain mutants. TCL shows the levels of endogenous PARP1. Total GST-Smad1 mutant proteins used for immunoblotting of endogenous PARP1 are stained with Coomassie Brilliant Blue in the *middle panel*. The Smad1 sequence motif that was mutated (*red letters*) and that represents a genuine ADP-ribosylation target sequence is shown in the *bottom panel*. A representative immunoblot of three repeats is shown. Molecular size markers in kDa are also marked. *D*, *in vitro* ADP-ribosylation assay of GST-Smad1-MH1 domain mutants. Control GST, beads, WT-Smad1-MH1 domain, and three mutants (as shown in *C*) were incubated with ^32^P-β-NAD^+^ and recombinant PARP1. ADP-ribosylated proteins were imaged via autoradiography. The radioactive protein bands of PARP1 and GST-Smad1-MH1 are marked. Total GST proteins were checked by Coomassie Brilliant Blue staining. *Lane 1/3 WT* indicates a reaction where one-third of the GST-Smad1-MH1 protein was used compared with the WT lanes. A representative autoradiogram of two assays is shown. Molecular size markers in kDa are also marked. *E*, immunoblot of recombinant PARP1 (20 ng) bound to the indicated GST-Smad1 MH1 domain mutants. The experiment is a repeat of the ribosylation assay of Fig. 8*D*, except that only cold β-NAD^+^ was used during incubation, followed by pulldown and immunoblotting. On the side, increasing amounts of recombinant PARP1 along with TCL from HEK293T cells show the levels of recombinant PARP1 used in the assay relative to endogenous PARP1. Total GST-Smad1 mutant proteins checked by Coomassie Brilliant Blue staining, used for immunoblotting of recombinant PARP1. A representative immunoblot of two repeats is shown. Molecular size markers in kDa are also marked. *F*, molecular model adapted to a detail from the crystal structure of two Smad3 MH1 domains bound to the Smad-binding DNA element (PDB code 1mhd). Shown is a ribbon diagram of the whole Smad3 MH1 domain with colored amino acids and the acceptor glutamate (*red*) and lysine (*blue*) residues drawn as stick and ball structures on the bottom side of the surface of the regulatory α-helix of one Smad3 MH1 subunit (*white arrow*). The β-hairpin that contacts DNA is also indicated (*white arrow*). *WB*, Western blotting.

We then asked whether the interaction between Smad1 or Smad5 and PARP1 led to catalytic modification of the Smads via ADP-ribosylation (Fig. 8*A*). Using an *in vitro* ADP-ribosylation assay based on recombinant proteins, we could demonstrate robust ADP-ribosylation of both Smad1 and Smad5 (Fig. 8*A*). The ADP-ribosylation of Smad1 and Smad5 was significantly weaker compared with Smad3 (compare the radioactive ADP-ribose signal to the total amount of protein used as substrate (Coomassie Brilliant Blue staining)) (Fig. 8*A*). Note that because the ADP-ribosylation is performed using GST fusions of Smads, the assay is performed as a pulldown, which allows us to confirm that ADP-ribosylated PARP1 (auto-catalyzed) is bound to its substrate, Smad1 or Smad5.

Including recombinant PARG in the ADP-ribosylation reaction showed that PARG de-ADP-ribosylated both PARP1 and Smad1 or Smad5 (Fig. 8*B*). Based on our previous study of ADP-ribosylation of Smad3 and Smad4, we hypothesized that Smad1 was ADP-ribosylated at the homologous amino acid motif to that in Smad3 (22). The conserved motif has the form of EELEK in Smad1 (Fig. 8*C*). This motif lies near the β-hairpin structure that binds directly to DNA and modulates the DNA binding activity of Smad1 (Fig. 8*F*). Mutating Glu^49^, Glu^50^, and Glu^52^ to Gln or the adjacent Lys^53^ to Arg did not perturb the association of these mutant Smad1 MH1 domains with endogenous PARP1 from HEK293T cells (Fig. 8*C*), confirming that point mutations in this α-helical part of the MH1 domain did not perturb the overall folding of the domain in a manner that would perturb association with PARP1. The Glu^49^, Glu^50^, and Glu^52^ to Gln mutations reduced the ADP-ribosylation of wild-type Smad1 MH1 domain by roughly 35% (Fig. 8*D*). Mutating Lys^53^ to Arg also showed a corresponding 33% reduction in ADP-ribosylation relative to wild type. Mutating Lys^53^ to Ala resulted in a more serious loss of ADP-ribosylation that was almost background (Fig. 8*D*). This mutant interacted well with endogenous PARP1 (Fig. 8*E*) and with the recombinant PARP1 (Fig. 8*E*), when compared with the other point mutants and the wild-type MH1 domain. We therefore conclude that Smad1 is ADP-ribosylated in the short EELEK motif that precedes the β-hairpin domain (Fig. 8*F*), a site that is equivalent to the ADP-ribosylation site in Smad3 (22). Both Glu and Lys residues appear to be acceptor sites for ribosylation in the MH1 domain of Smad1.

##### PARP1 Inhibition Has an Impact on Physiological Responses to BMP Signaling during Differentiation

**FIGURE 9.**
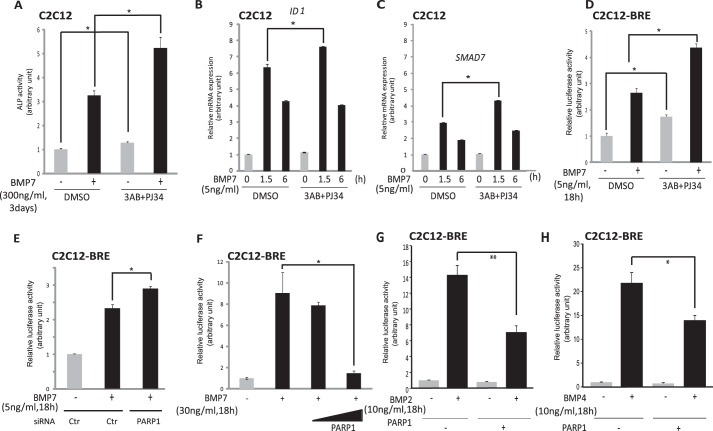
**Inhibition of endogenous ADP-ribosylation activity promotes physiological responses to BMP7.** *A*, alkaline phosphatase assay to assess myoblast to osteoblast differentiation in C2C12 mouse cells, treated with 5 mm 3-AB and 3 μm PJ34 (ADP-ribosylation inhibitors), and then stimulated with vehicle or 300 ng/ml BMP7 for 3 days. *Bars* represent the relative alkaline phosphatase activity as a mean value from triplicate determinations and standard deviation is shown with *error bars*. The measurements were normalized to total protein levels. *Asterisks* indicate a statistically significant differences at *p* < 0.05. *B* and *C*, qRT-PCR for *ID1* (*B*) and *SMAD7* (*C*) in lysates from C2C12 cells treated with the chemical ADP-ribosylation inhibitors 3-AB and PJ34 at 5 mm and 3 μm concentrations, respectively, or with vehicle (DMSO), 1 h prior to stimulation with 5 ng/ml BMP7 or vehicle for the indicated time periods. The data are presented as in Fig. 1*A*. An *asterisk* indicates a statistically significant difference at *p* < 0.05. *D*, luciferase reporter assay in mouse C1C12 myoblasts, stably transfected with the BRE_2_-luciferase reporter construct (C2C12 BRE-luc cells) treated with the ADP-ribosylation inhibitors 5 mm 3-AB and 3 μm PJ34 or with vehicle (DMSO), 1 h prior to stimulation with 5 ng/ml BMP7 or vehicle for 18 h. The data are presented as in Fig. 2*C. Asterisks* indicate a statistically significant difference at *p* < 0.05. *E*, luciferase reporter assay in C2C12 BRE-luc cells transiently transfected with siRNA targeting PARP1 (siPARP1) or non-targeting siRNA (*siCtr*) and in the presence or absence of stimulation with 5 ng/ml BMP7 for 18 h. The data are presented as in Fig. 1*E*. An *asterisk* indicates a statistically significant difference at *p* < 0.05. *F*, luciferase reporter assay in C2C12 BRE-luc cells upon transient transfection of increasing doses of myc-PARP1 or a control empty vector (pcDNA3) and in the absence or presence of 30 ng/ml BMP7 for 18 h. The data are presented as in Fig. 2*C*. An *asterisk* indicates a statistically significant difference at *p* < 0.05. *G*, luciferase reporter assay in C2C12 BRE-luc cells upon transient transfection of myc-PARP1 (+) or a control empty vector (pcDNA3, −) and in the absence or presence BMP2 for 18 h. The data are presented as in Fig. 2*C*. An *asterisk* indicates a statistically significant difference at *p* < 0.05, and *double asterisks* indicate a statistically significant difference at *p* < 0.01. *H*, luciferase reporter assay in C2C12 BRE-luc cells upon transient transfection of myc-PARP1 (+) or a control empty vector (pcDNA3, −) and in the absence or presence BMP4 for 18 h. The data are presented as in Fig. 2*C*. An *asterisk* indicates a statistically significant difference at *p* < 0.05, and *double asterisks* indicate a statistically significant difference at *p* < 0.01. *ALP*, alkaline phosphatase.

**FIGURE 10.**
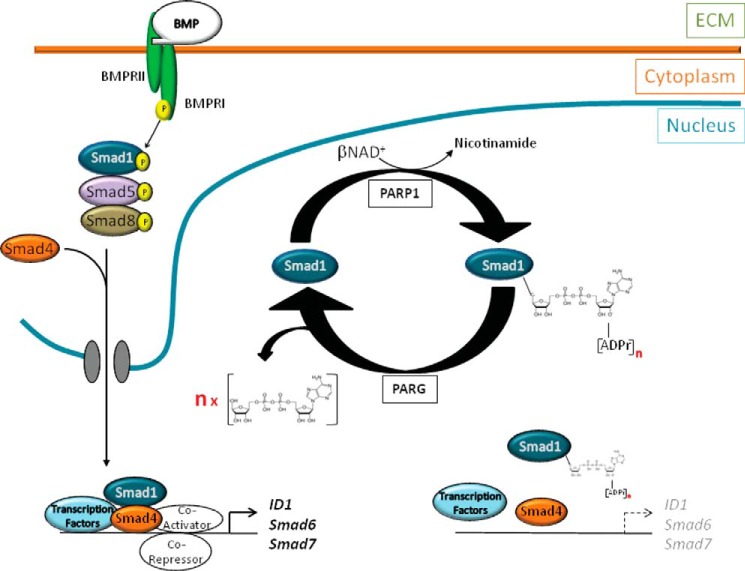
**PARP1 and PARG modulate Smad1 activity during BMP signaling.** A model shows the BMP ligand activating cell surface receptors II and I, with the latter phosphorylating Smad1, Smad5, and Smad8, facilitating oligomerization with Smad4 to enter the nucleus through a nuclear pore. The nuclear Smad complex associates with transcription factors, co-activators or co-repressors on chromatin to regulate target genes such as *ID1*, *SMAD6*, and *SMAD7*. The ADP-ribosylating enzyme PARP1 interacts with the nuclear Smad complex. With β-NAD^+^ as a donor, PARP1 attaches ADP-ribose chains (of *n* size, [ADPr]*_n_*) on itself (not shown) and on to the Smad complex to assist its dissociation from DNA or inhibit association with DNA (Smad proteins away from DNA). PARG that has affinity toward ADP-ribose chain promotes de-ADP-ribosylation resulting in mono-ADP-ribose chains on the Smad complex (not verified experimentally and not shown for simplicity) and a pool (*n*) of ADP-ribose, thus facilitating enhanced binding of the Smad complex to DNA. For simplicity, the figure emphasizes Smad1 ADP-ribosylation. The extracellular matrix (*ECM*), cytoplasmic, and nuclear cell compartments are highlighted.

To confirm the functional relevance of the above findings of Smad1/5 ADP-ribosylation by PARP1, we relied on well established chemical inhibitors of this enzyme. In C2C12 pluripotent mesenchymal cells, induction of alkaline phosphatase, an established marker of osteoblastic differentiation (8), was strongly induced by BMP7 stimulation, and inhibition of PARP1 catalytic activity reproducibly enhanced the BMP7-mediated osteoblast differentiation (Fig. 9*A*). In a similar manner, catalytic inhibition of PARP1 with two chemical inhibitors, 3-amino-benzamide and PJ-34, also enhanced the BMP-dependent expression of *ID1* and *Smad7* (Fig. 9, *B* and *C*). Similar to the effects on endogenous gene regulation, the inhibition of PARP1 catalytic activity with the two chemical inhibitors, 3-amino-benzamide and PJ-34, resulted in significantly enhanced transcriptional signaling by BMP7 in the C2C12-BRE-luc cells (Fig. 9*D*). Replacing the chemical inhibitor with genetic interference using the PARP1 siRNA showed that silencing of endogenous PARP1 in the C2C12-BRE-luc cells reproducibly enhanced BMP7 signaling at the level of the BRE_2_-luc reporter activity (Fig. 9*E*). Finally, the inverse experiment with overexpression of wild-type PARP1 in the C2C12-BRE-luc cells followed by 18 h of stimulation with BMP7 led to robust activation of the reporter, and co-transfection of wild-type PARP1 reduced the reporter activity almost to background levels (Fig. 9*F*). The same negative effect of PARP1 overexpression was also verified when the transcriptional reporter was activated by BMP2 or BMP4 instead of BMP7 in the same cell model (Fig. 9, *G* and *H*). This finding attests that the impact of PARP1 on BMP-specific Smad signaling is independent from the specific ligand that activates the Smad pathway. Our overall conclusion is that PARP1 and PARG associate with BMP-specific Smad proteins and regulate their transcriptional output in opposing ways, whereby PARP1 acts as a negative and PARG as a positive regulator of gene expression downstream of BMP (Fig. 10).

## Discussion

This study comes as a natural follow up of a series of recent reports that established for the first time that enzymes of the ADRT family, namely PARP1, PARP2, and PARG, regulate the flow and biological outcome of signal transduction by TGF-β (22–25, 27). Because BMP pathways have distinct biological and molecular functions, we focused on the regulation of BMP signaling by PARP1 in the present paper. The current experimental evidence presents a new molecular regulatory model for the BMP pathway (Fig. 10). Because BMP-specific Smad complexes enter the nucleus and associate with chromatin to regulate gene expression, they also encounter the action of PARP1 and PARG. PARP1 ADP-ribosylates Smad1, Smad5, and Smad4, whereas PARG de-ADP-ribosylates these Smads, and this dynamic post-translational modification has an impact on gene regulation and cell differentiation (Fig. 10).

Our previous work on Smad3 proposed that ADP-ribosylation of the MH1 domain motif negatively impacts DNA binding by Smad3 (22). A similar mechanism may operate at the level of BMP R-Smads. This is compatible with all effects measured on the regulation of several endogenous genes that are established targets of BMP signaling (Figs. 1, 2, 4, 5, and 9). PARG acts as a positive and PARP1 as a negative regulator of gene expression and osteoblast differentiation (Fig. 9). The magnitude of effects measured when endogenous PARP1 or PARG are inhibited either via RNAi or via pharmacological intervention are reproducible and significant but do not exhibit all-or-nothing behavior. We interpret this finding as an indication that PARP1 and PARG act as intermediate regulators of nuclear Smad activity that is interwoven together with other site-specific enzymatic activities, *e.g.* nuclear phosphorylation, dephosphorylation, acetylation, monoubiquitylation, and polyubiquitylation, that collectively regulate the activity of the chromatin-bound Smad complexes. An example of such a BMP Smad co-factor that attracts PARP family enzymes and contributes to the full biological outcome of BMP signaling is the known multizinc finger transcriptional regulator OAZ (31). When PARP1 is bound to OAZ, it positively promotes transcriptional regulation induced by BMPs in P19 teratocarcinoma cells, suggesting that the presence or absence of OAZ in nuclear complexes of BMP Smads may define the direction, magnitude, and specificity of regulatory events catalyzed by PARP1 (31). In future work, it would be interesting to examine the specific window in the time course of nuclear Smad functions during which ADP-ribosylation becomes most critical. The PLA analysis (Fig. 7, *D* and *E*) suggests that this time window will be relatively early; however, this may differ from cell type to cell type and possibly even under different pathophysiological conditions (27, 31).

By analyzing the interaction of Smad1 with PARG, we identified a relative selectivity of this association, whereby Smad1 seems to fail to form complexes with PARG, whereas Smad5 (and Smad4) form readily detectable complexes at the endogenous level or upon overexpression (Fig. 3). At the moment, we do not understand the reason behind this selectivity. In cells, because BMP signaling organizes multi-Smad complexes consisting of Smad1, Smad5, and Smad4, it is possible that the presence of one Smad subunit suffices for bringing PARG into the nuclear Smad complex. However, the selectivity observed in the co-immunoprecipitation assays must have another structural and possibly biological reason that needs to be investigated deeper. The investigation of the subcellular localization of PARG-Smad4 complexes aimed to clarify whether nuclear or cytoplasmic PARG isoforms participate in the formation of protein complexes with Smad4 and revealed only nuclear localization of the complexes. Taking into account that cytoplasmic PARG isoforms have catalytic activity, the observation of the nuclear PARG-Smad4 complex possibly implies a rapid de-ADP-ribosylation of Smad4, concomitant to its ADP-ribosylation by PARP1.

The BMP-specific Smad1 and Smad5 form complexes with PARP1 (Fig. 7). The MH1 domain of Smad1, similar to Smad3 and Smad4, provides the specificity for this interaction (Fig. 7*F*), which is compatible with the fact that specific amino acids in the MH1 domain become ADP-ribosylated by PARP1 (Fig. 8). On the other hand, it is also possible that the Smad1 MH1 domain association with DNA regulates the interaction with PARP1 and the subsequent ADP-ribosylation. It is of course formally possible that PARP1 meets Smad1 prior to its association with DNA; however, growing evidence from genome-wide screens and deeper understanding of the functions of chromatin regulators like PARP1 suggest that most of these intermolecular associations and post-translational modifications occur on chromatin. Possible development of *in situ* PLA coupled with fluorescent *in situ* hybridization technology (32) may allow us to resolve this interesting question. In many of the interaction assays, we were able to monitor Smad1-PARP1 complexes in the absence of BMP stimulation; however, BMP stimulation or PARP1 activation by means of peroxide treatment both significantly enhanced the abundance of complexes formed (Fig. 7). Treating cells with a brief pulse of peroxide is sufficient to activate endogenous PARP1 and very quickly this leads to an enhanced complex with Smad1 and Smad5 (Fig. 7*A*). Although it is possible that peroxide might affect other molecular players in the cell (*e.g.* protein phosphatases), our collective evidence supports a PARP1-dependent role that enhances the association between Smads and PARP1. Using PLA, we identified a rapid kinetic profile of Smad1-PARP1 complex formation at the endogenous level (Fig. 7, *D* and *E*). Although the majority of complexes measured were nuclear, PLA also revealed endogenous cytoplasmic complexes (Fig. 7*E*). Smad1 constantly shuttles in and out of the nucleus, whereas PARP1 shows more stable association with the nuclear compartment. Whether Smad1 carries PARP1 at different subcellular compartments at this stage is speculative and requires further deeper investigation. Similarly, the exact meaning of the dynamic changes of Smad1-PARP1 complexes over time of BMP signaling remains open to future analysis.

By analyzing the ADP-ribosylation of Smad1 and Smad5 *in vitro*, we found that both R-Smads can be modified by PARP1, and PARG can remove ADP-ribose chains from these Smads (Fig. 8). The motif identified as a putative ADP-ribosylation site in Smad1, EELEK, is well conserved among all Smad proteins and resides in the MH1 domain and juxtaposes the β-hairpin of the MH1 domain that binds to DNA (1–3). Mutating specific glutamate residues indicated a significant decrease of ADP-ribosylation but not complete loss (Fig. 8*D*). Mutation of Lys^53^ to Arg decreased the ADP-ribosylation efficiency, but its mutation to Ala failed to show ADP-ribosylation (Fig. 8*D*). The various MH1 domain point mutants employed here were synthesized and semipurified from *E. coli* (Fig. 8, *A–E*) and were capable of associating with endogenous (Fig. 8*C*) and recombinant (Fig. 8*E*) PARP1, suggesting that the mutations did not alter folding of the MH1 domain. Thus, Smad1 (and by extrapolation Smad5) may accept ADP-ribose units on the glutamate or lysine residues of the conserved EELEK motif within the MH1 domain, which is compatible with the general knowledge of ADP-ribosylation sites (17). PARG was capable of reducing the level of ADP-ribosylation of the Smad1 MH1 domain (Fig. 8*B*) but did not completely remove ADP-ribose from Smad1/5, even after prolonged incubation with PARG (Fig. 8*B*). Possibly, members of the ADP-ribosylhydrolase and macrodomain-containing protein families (19) may cooperate with PARG to achieve complete removal of ADP-ribosyl chains from the BMP-specific R-Smads.

We have established ADP-ribosylation and the actions of PARP1 and PARG as important regulatory steps in the progression and execution of physiological BMP signaling. These new findings also propose that PARP1 and PARG chemical modulators (*e.g.* inhibitors) (18, 33) could be of potential use to regulate the potency of the BMP pathways at will. This can be of importance for the control of BMP signaling activity in the many pathological cases where these pathways malfunction such as hypertension and cancer or various rare syndromes (11, 12). The recent finding that TGF-β sensitizes breast cancer cells to lethality in response to PARP1 inhibitors is fully compatible with our proposal (34). We therefore conclude that this investigation provides the necessary basic background based on which such future explorations can be founded.

## Author Contributions

Y. W. and A. M. conceived and designed the work; Y. W., P. P., V. M., and Y. T. acquired the data; Y. W., P. P., V. M., Y. T., M. O. H., C.-H. H., and A. M. analyzed and interpreted the data; and Y. W., P. P., V. M., C.-H. H., and A. M. drafted the article or revised it critically for important intellectual content. All authors approved the version of the paper to be published.

## Footnotes

5 Y. Watanabe, P. Papoutsoglou, V. Maturi, Y. Tsubakihara, M. O. Hottiger, C.-H. Heldin, and A. Moustakas, unpublished results.

## Footnotes

4 The abbreviations used are:

BMP: bone morphogenetic protein
PARP1: poly-(ADP-ribose) polymerase 1
PARG: poly-(ADP-ribose) glycohydrolase
R-Smad: receptor-activated Smad
I-Smad: inhibitory Smad
ARTD: ADP-ribosyltransferase structurally related to diphtheria toxin
MH1: Mad homology 1
PLA: proximity ligation assay
BRE: BMP-responsive element
MEF: mouse embryonic fibroblast
3-AB: 3-aminobenzamide
qRT-PCR: quantitative RT-PCR.
